# Evaluation of the thermal aging of δ-ferrite in austenitic stainless steel welds by electrochemical analysis

**DOI:** 10.1038/s41598-018-33422-x

**Published:** 2018-10-10

**Authors:** Gokul Obulan Subramanian, Byeong Seo Kong, Ho Jung Lee, Changheui Jang

**Affiliations:** 1 Korea Advanced Institute of Science and Technology, Daejeon, 34141 Republic of Korea; 2 Korea Hydro and Nuclear Power Co. Ltd., Daejeon, 34101 Republic of Korea

## Abstract

Cr-segregation by spinodal decomposition and G-phase precipitation were observed in δ-ferrite of austenitic stainless steel welds thermally aged at 400 °C for up to 20,000 h. A reversion heat treatment (R-HT) at 550 °C for 1 h dissolved the Cr-segregation in the aged welds while some intermetallic precipitates were present. The double-loop electrochemical potentiokinetic reactivation (DL-EPR) analysis showed no significant differences among them. However, after selective etching of the austenite phase, the DL-EPR values of δ-ferrite phase steadily increased with aging time due to the growth of Cr-depleted regions by spinodal decomposition. The electrochemical behavior of δ-ferrite after R-HT condition was similar to that of unaged welds, indicating the intermetallic precipitates did not affect the corrosion resistance in this case. Overall, DL-EPR analysis of δ-ferrite phase provided better correlation with spinodal decomposition.

## Introduction

Austenitic stainless steels are widely used as coolant pipes in light water reactors and pressurizer surgeline pipes in pressurized water reactors (PWR), which are typically joined by fusion welding methods. The resulting weld microstructure has austenite matrix, where typically 4–12% of δ-ferrite is left in the weld fusion zone to avoid hot cracking^1^. The welds are usually exposed to temperatures between 290–343 °C in PWR systems during operation and subjected to microstructural evolution. It is susceptible to thermal aging embrittlement, resulting in an increase in hardness and strength in compromise of a reduction in ductility and fracture toughness^2–4^.

The thermal aging during operation of light water reactors has been known to be related to phase separation, where the δ-ferrite decomposes into nano-sized Fe-rich α and Cr- rich α’ phases^1–5^. The phase separation in δ-ferrite either occurs by spinodal decomposition or nucleation and growth, depending upon the alloy composition and temperature. In general, the δ-ferrite in welds decomposes by spinodal decomposition^1–11^, with mottled ferrite appearance and intertwined structure. The precipitation of Ni- and Si-rich G-phase and Cr-rich M_23_C_6_ carbide is also reported to happen during thermal aging in welds^2,3^. The phase separation resists dislocation movement resulting in thermal embrittlement, while G-phase contributes to some extent based on the alloy composition and aging time^9,12–16^. A reversion heat treatment (R-HT) can be applied to recover the mechanical properties degraded due to phase separation by dissolving it, while the residual intermetallic precipitates are left in the δ-ferrite microstructure^9,14–16^.

Due to spinodal decomposition, the Cr-content in Fe-rich α phase will be depleted significantly, resulting in the degradation of corrosion resistance of δ-ferrite phase. Many researchers have utilized the electrochemical analysis to study the phase separation of ferrite in thermally aged duplex stainless steels^17–36^, ferritic stainless steels^37–40^ and austenitic stainless steel welds^6–8,10,11^. In general, the corrosion resistance has been observed to degrade with the evolution of phase separation over long aging periods, where the Cr-depleted, Fe-rich α phase is more susceptible towards electrochemical dissolution. It is also reported that, the precipitation of other intermetallic phases like G-phase together with the phase separation affects the corrosion resistance^6–8,10,11,25,34–36,39^.

It is well known that the austenite and ferritic phase show independent electrochemical responses at different electrochemical potentials^17,41–43^. For instance, Jiang *et al*.^17^ observed separate activation/reactivation peaks corresponding to ferrite and austenite phases in a duplex stainless steel containing 65% ferrite content, where the reactivation current density of the ferrite was found to increase with aging time. In the case of austenitic stainless steels containing smaller ferrite fraction, the electrochemical response of ferrite will be completely overlapped with that of austenite in the double-loop electrochemical potentiokinetic reactivation (DL-EPR) analysis, without any indications of ferrite electrochemical response^6–8,10,11,19,33^. Further, the ferrite remains in the passivation regime at the point where austenite is electrochemically active at higher electrochemical potentials. In such cases, the electrochemical parameters for studying the thermal aging were taken from the region where austenite is electrochemically active, therefore compromising the electrochemical responses from the ferrite phase where the nano-scaled phase separation has occurred. Since much of the microstructural changes associated with the thermal embrittlement originates from the ferrite matrix^5,6,29,30,32,44^, it is important to separate the ferrite electrochemical responses from that of austenite matrix, to precisely associate the electrochemical behavior changes with thermal embrittlement. Park *et al*.^21^ used micro-capillary technique while Örnek *et al*.^34^ used scanning Kelvin probe force microscopy to study the electrochemical behavior ferrite phase in aged duplex stainless steels. However, these techniques would not be applicable for conventional electrochemical analysis of ferrite phase in aged stainless steels.

To address this concern, we have studied the DL-EPR analysis of δ-ferrite exclusively by selectively etching the austenite matrix. Then, we have compared the electrochemical response of the weld material (termed as bulk material) as used in the reported studies^6–8,10,11^, with that of the δ-ferrite phase only after etching the austenite. Further, we have discussed about the implications of studying the electrochemistry of δ-ferrite phase only compared to the bulk material. Also in this study, we have dissolved the phase separation in δ-ferrite using R-HT leaving only intermetallic precipitates, allowing us to study its effect on the corrosion resistance of aged materials.

## Results and Discussions

### TEM microstructural analysis

**Figure 1:**
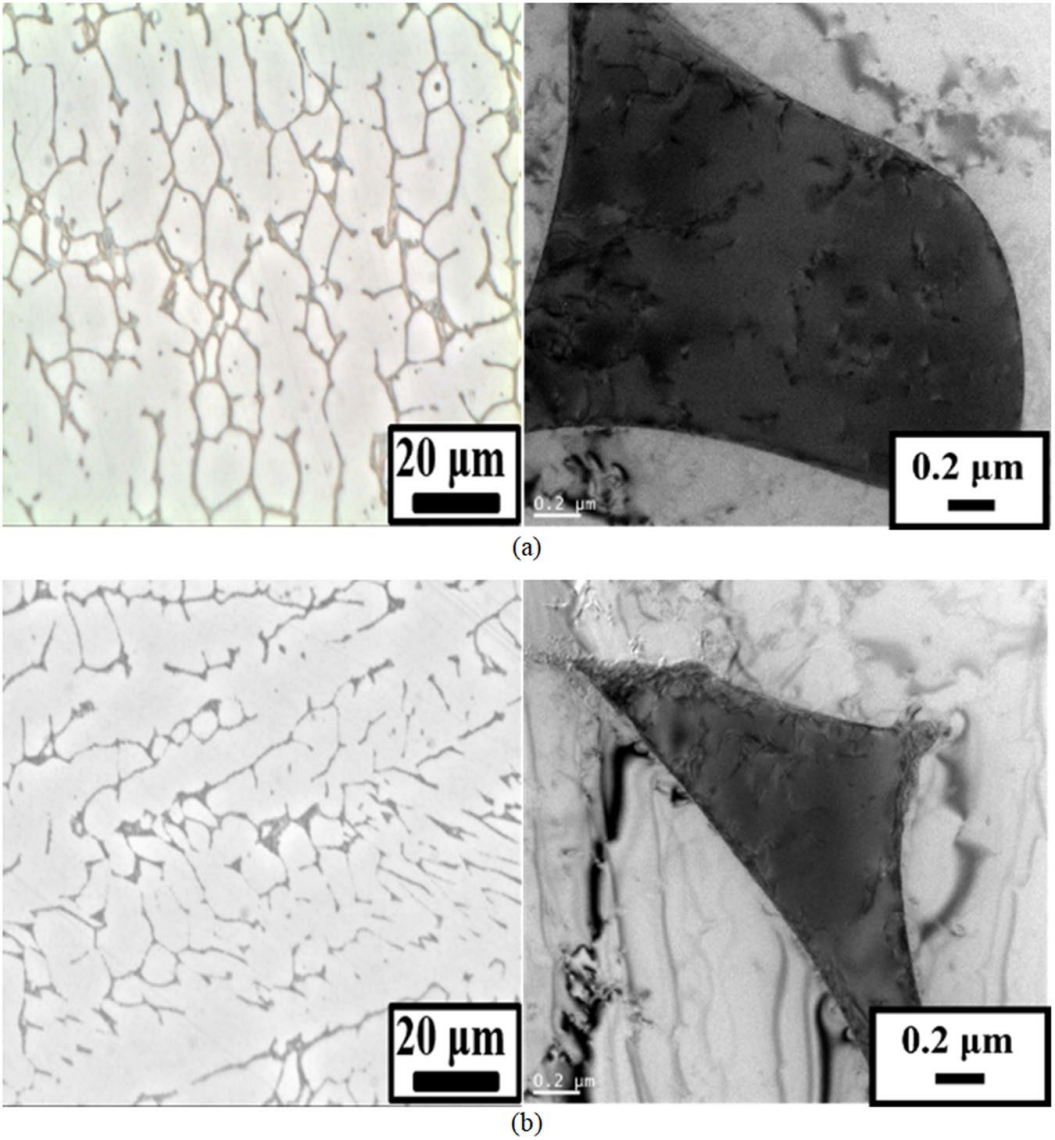
Optical and TEM micrographs of (**a**) ER316L, and (**b**) ER347 in as-welded condition.

Figure 1 shows the optical and transmission electron microscopy (TEM) micrographs of the as-welded ER316L and ER347 welds. Very thin (mostly less than 1 μm width) δ-ferrites were developed along the dendrite direction for both welds, indicated by the darker phase in Fig. 1. Based on the TEM microstructural analysis (not shown here) Cr was enriched in δ-ferrite phase, while Ni and Mn were enriched in the austenite matrix. In addition, Mo was found to be rather evenly distributed in δ-ferrite phase and austenite matrix in ER316L weld. In the as-welded condition of ER347 weld, fine NbC precipitates were additionally present mostly along the δ-ferrite/austenite phase boundaries. The authors previously observed that for ER347 weld aged at 400 °C had smaller domain sizes compared to the aging treatment at 450 °C (not shown here). Also, the compositional fluctuation increase was gradual corresponding to the uphill diffusion by spinodal decomposition.

**Figure 2:**
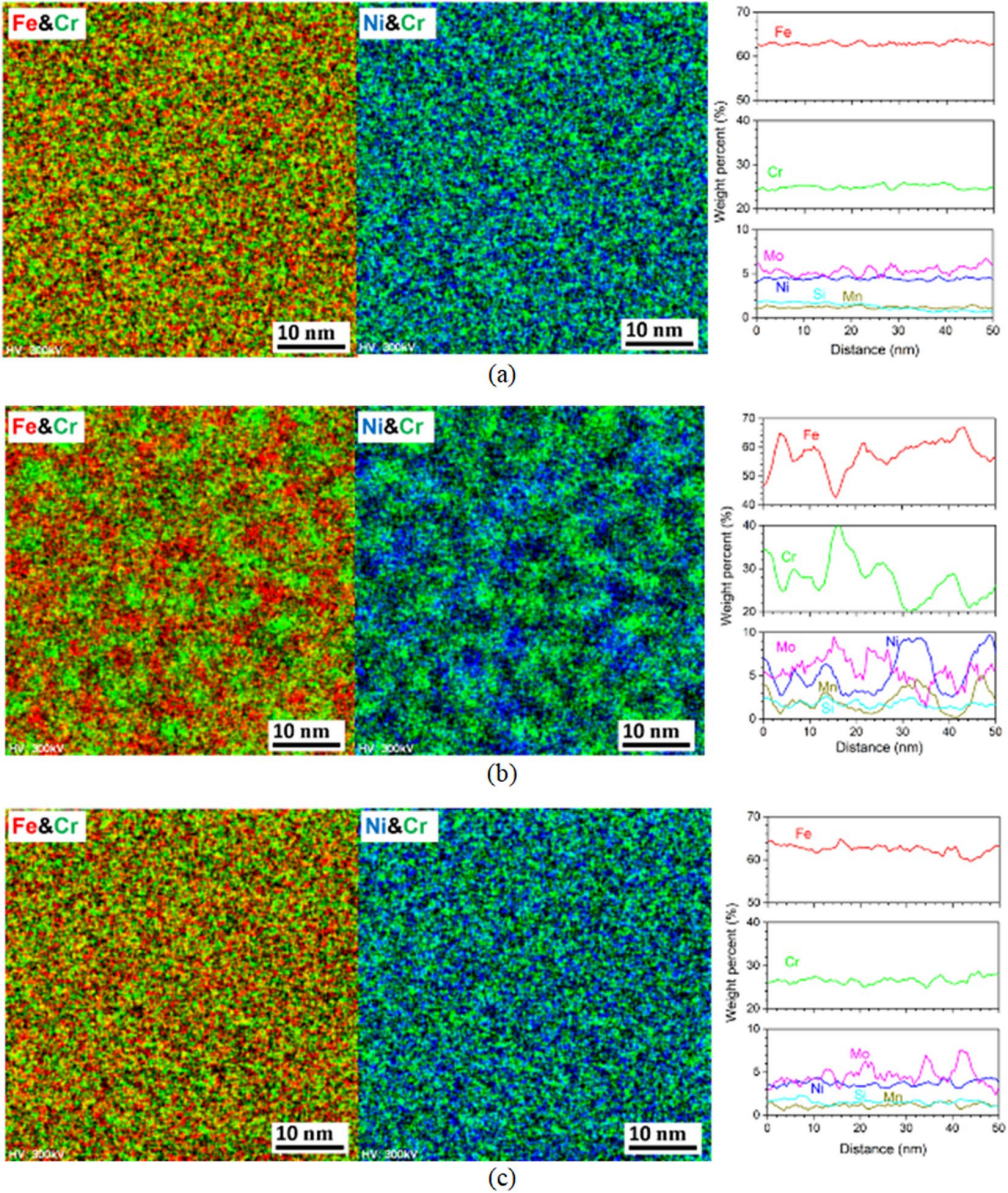
STEM/EDS mapping and line scan results of δ-ferrite in ER316L weld (**a**) as-welded condition, (**b**) aged at 400 °C for 20,000 h, and (**c**) R-HT condition.

**Figure 3:**
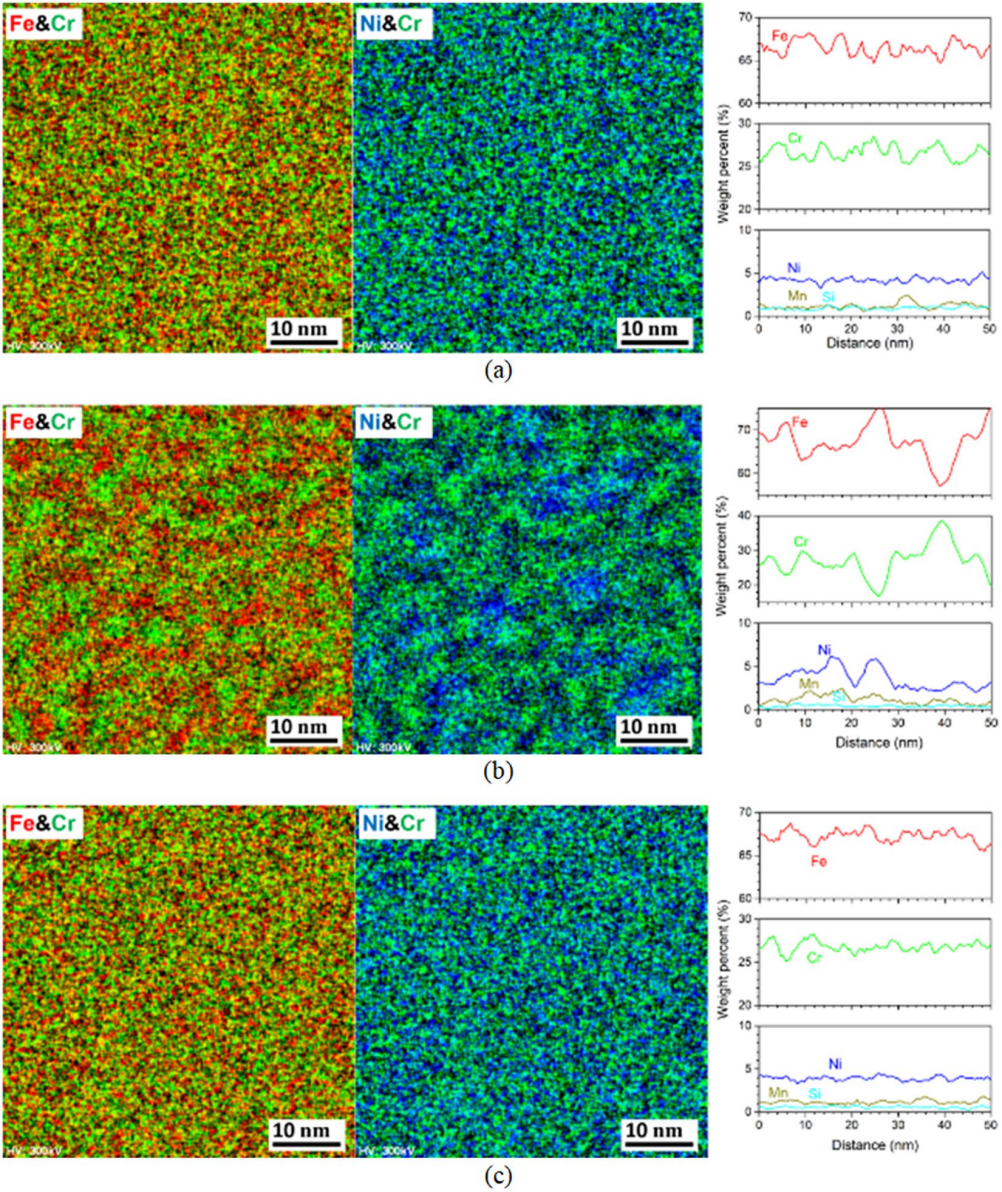
STEM/EDS mapping and line scan results of δ-ferrite in ER347 weld (**a**) as-welded condition, (**b**) aged at 400 °C for 20,000 h, and (**c**) R-HT condition.

Figures 2 and 3 show scanning transmission electron microscopy - energy dispersive X-ray spectroscopy (STEM/EDS) analysis results of δ-ferrite phase in ER316L and ER347 welds respectively, for the as-welded, aged (400 °C for 20,000 h), and R-HT conditions. In the as-welded condition (Figs 2a and 3a), alloying elements were distributed rather homogeneously in δ-ferrite phase, with an average Cr-content of 25.0 wt.% and 26.6 wt.% for ER316L and ER347 welds, respectively. After thermal aging at 400 °C for 20,000 h (Figs 2b and 3b), significant fluctuation in element concentration is observed. The concentration profiles show segregation of Fe- and Cr-rich domains, which corresponds to the phase separation of δ-ferrite to α and α’ phases by spinodal decomposition. From the STEM/EDS elemental mapping, α and α’ regions have a width of approximately 5 nm. Furthermore, from the STEM/EDS line scan profiles, Ni and Mn appeared to be segregated in α phase. In addition, Mo appeared to be segregated in the Cr-rich α’ phase after thermal aging in ER316L weld. The fluctuation in Cr content ranged from 19.7 to 40.3 wt.% for ER316L weld, while it ranged from 16.6 to 38.7 wt.% for ER347 weld. The measured fluctuation in element concentration is summarized in Tables 1 and 2.

**Table 1:** Summary of fluctuation of element concentration in the δ-ferrite in ER316L weld.

Condition	Cr		Fe		Ni	
	Min.	Max.	Min.	Max.	Min.	Max.
As-welded	24.09	25.99	62.23	63.85	3.99	4.89
400 °C for 20,000 h	19.70	40.32	42.53	66.91	2.55	9.76
400 °C for 20,000 h + R-HT	24.80	28.64	59.91	64.74	3.07	4.41

**Table 2:** Summary of fluctuation of element concentration in the δ-ferrite in ER347 weld.

Condition	Cr		Fe		Ni	
	Min.	Max.	Min.	Max.	Min.	Max.
As-welded	25.22	28.52	64.69	68.21	3.21	5.13
400 °C for 20,000 h	16.66	38.67	57.16	76.12	2.08	6.16
400 °C for 20,000 h + R-HT	25.18	28.24	65.48	68.78	3.29	4.51

**Figure 4:**
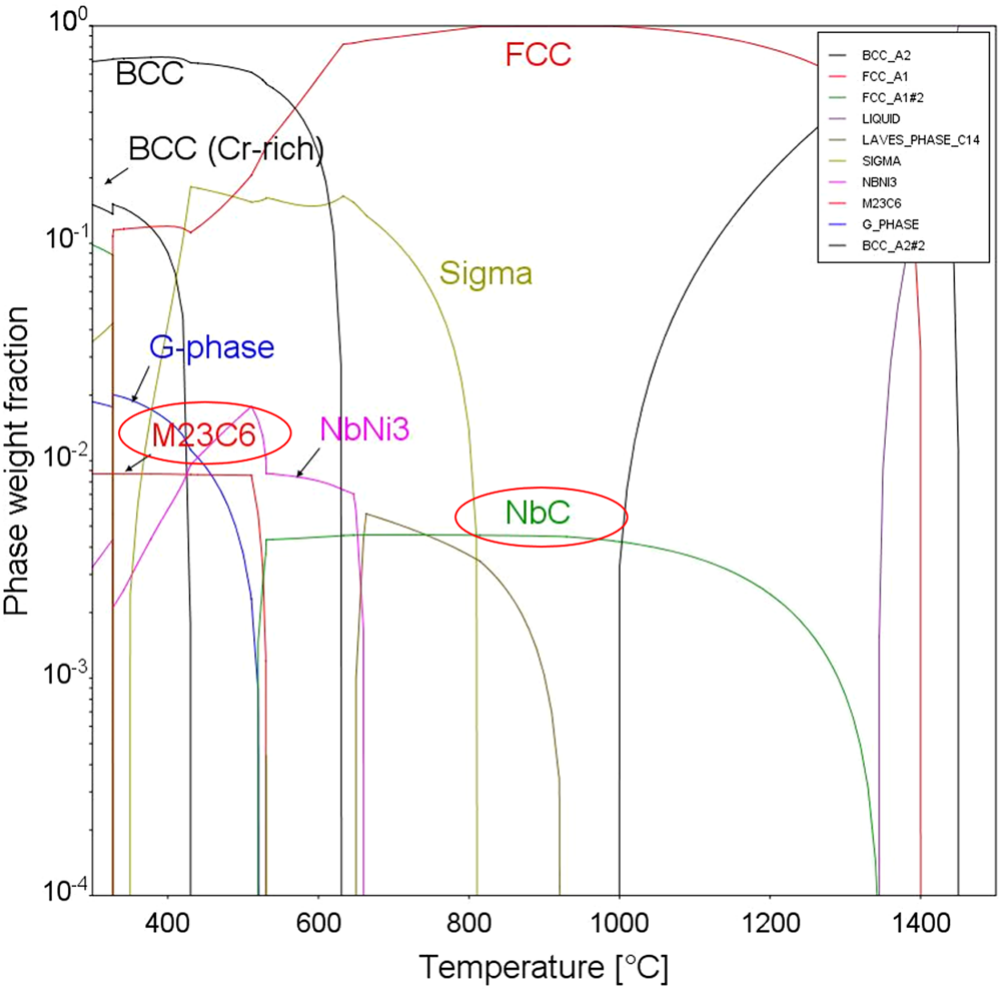
Thermo-Calc (TCFE-9 database) phase stability diagram for ER347 weld.

In our previous studies, no significant differences in concentration fluctuation was observed between the specimens thermally aged for 5,000–20,000 h from the STEM/EDS line scan analysis of focused ion beam (FIB) prepared TEM specimens, due to limitations in TEM resolution (not shown here). Nonetheless, it was reported that using atom probe tomography (APT) on aged welds, they have observed slower growth rate during longer aging periods at 400 °C^45^. Meanwhile, the fluctuation in elemental concentration almost disappeared in R-HT conditions (Figs 2c and 3c), suggesting dissolution of α and α’ phase separation. Precipitation of M_23_C_6_ was not observed in ER316L weld, which can be attributed to lower C content (0.008 wt.%). Also for the ER347 weld, M_23_C_6_ precipitation was not observed from the TEM analysis. Figure 4 shows the Thermo-Calc phase diagram of ER347 weld as a function of temperature with TCFE9 database. It can be observed that, NbC is relatively stable at higher temperature, while M_23_C_6_ appears at lower temperature (<500 °C). Also, NbC and M_23_C_6_ are not stable together, where the stability of one carbide requires the dissolution of the other. To stabilize M_23_C_6_, it also requires the formation of NbNi_3_. In this study, the NbC in the aged condition (400 °C for 20,000 h) is similar to that of as-welded condition in terms of particle size and number density. Also, we did not observe any precipitation of NbNi_3_ phases.

**Figure 5:**
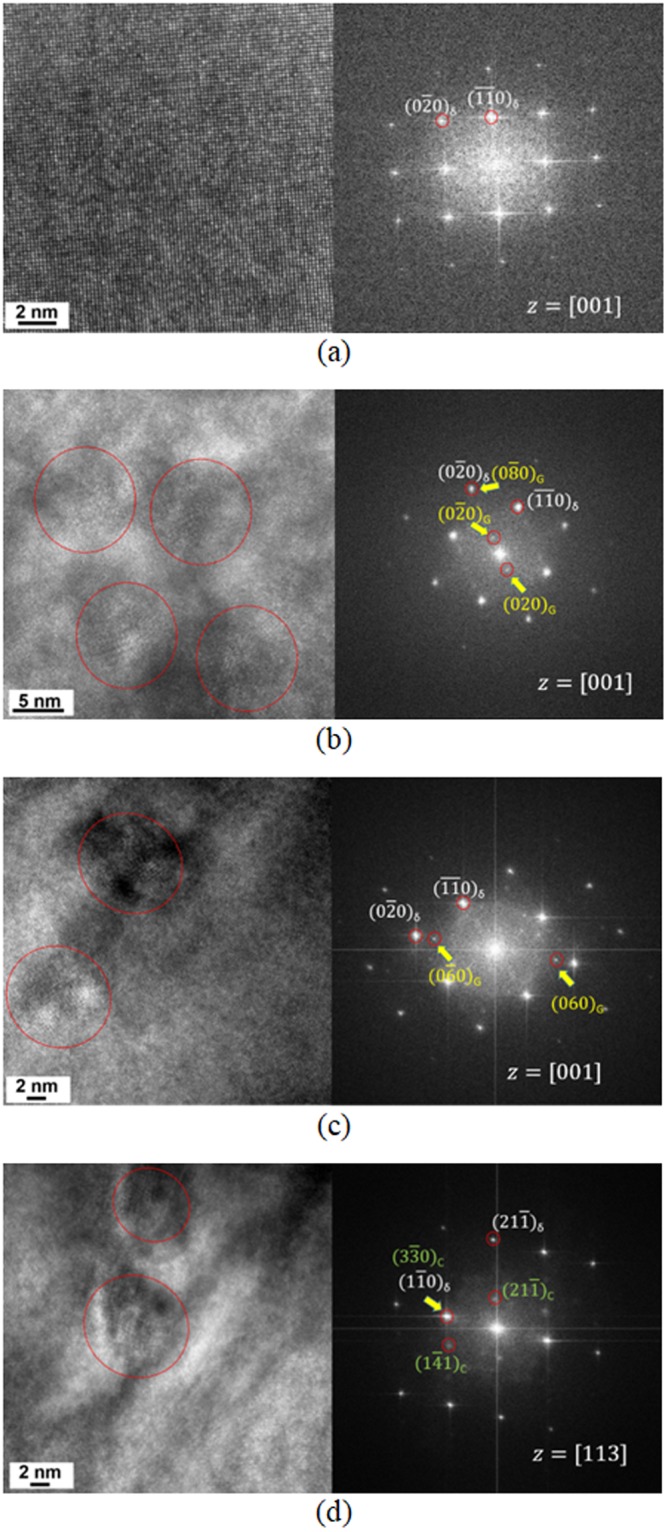
HR-TEM micrographs and FFT patterns of δ-ferrite in ER316Lweld (**a**) as-welded condition, (**b**) aged at 400 °C for 20,000 h, and (**c**) R-HT condition. The fine intermetallic precipitates observed in the HR-TEM micrograph are encircled.

**Figure 6:**
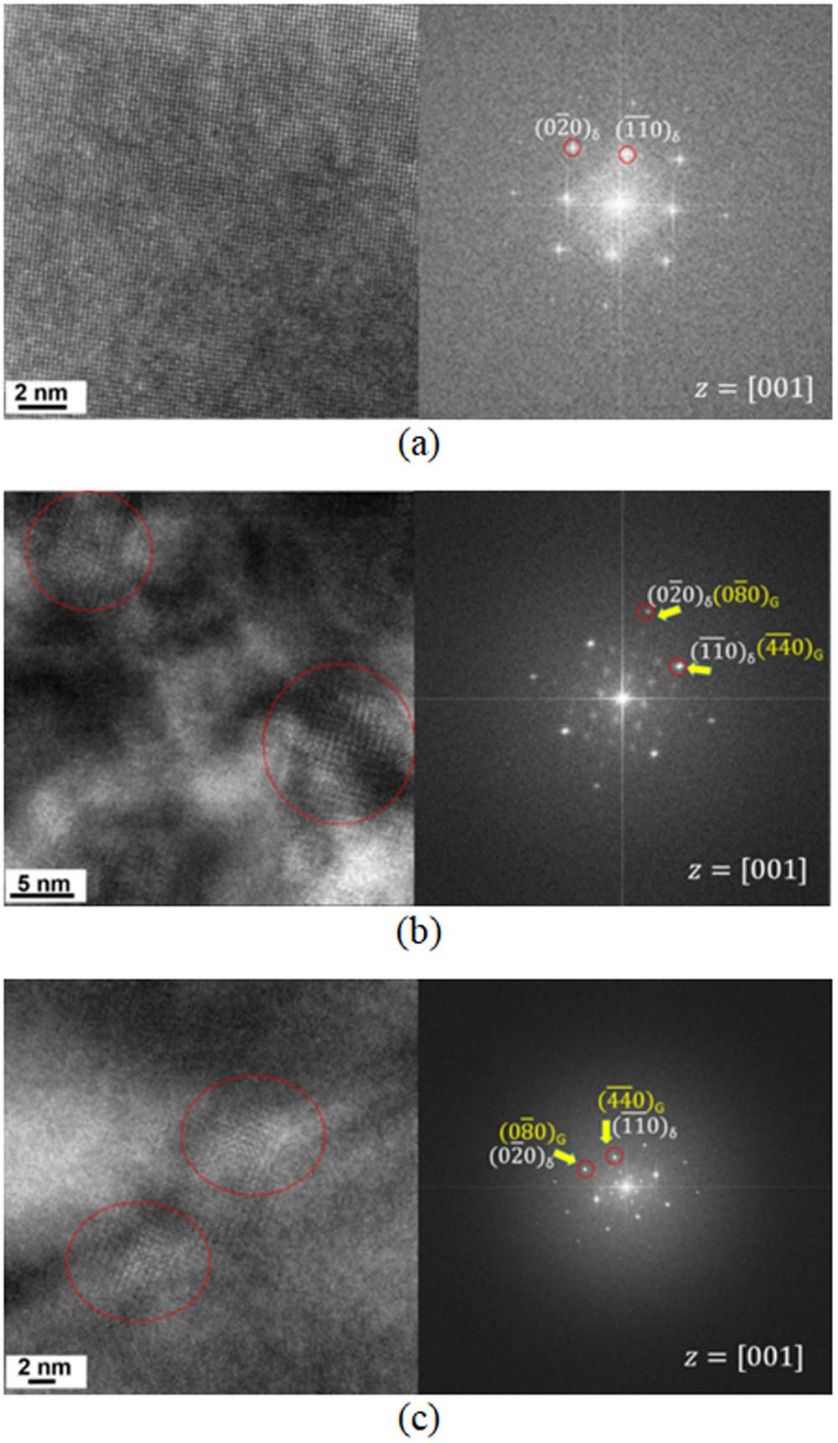
HR-TEM micrographs and FFT patterns of δ-ferrite in ER347weld (**a**) as-welded condition, (**b**) aged at 400 °C for 20,000 h, and (**c**) and (**d**) R-HT condition. The fine intermetallic precipitates observed in the HR-TEM micrograph are encircled.

Furthermore, high-resolution transmission electron microscopy (HR-TEM) analysis was conducted on the δ-ferrite of both welds to identify fine intermetallic precipitates. The HR-TEM lattice images and corresponding fast Fourier transformation (FFT) patterns are shown in Figs 5 and 6 for δ-ferrites in ER316L and ER347 welds respectively, in the as-welded, aged and R-HT conditions. The HR-TEM lattice image in the as-welded condition exhibited homogeneous ferrite matrix and the corresponding FFT pattern only showed spots of BCC phase. The FFT pattern of the aged specimens also showed FFT pattern of the BCC phase along [001] zone axis. Despite the separation of δ-ferrite to α and α’ phases by spinodal decomposition, no indications of lattice mismatch or strain were observed, maybe due to the resolution limitation at high magnification. Both aged ER316L and ER347 welds exhibited an additional FFT spots (marked yellow in FFT patterns in Figs 5b and 6b). The precipitates corresponding to these FFT spots are encircled in the HR-TEM lattice images, which are identified as G-phase with FCC structure (stoichiometrically (Ni,Fe)_16_(Mn,Cr)_6_Si_7_)^46^ with a lattice parameter of 1.14 nm. From the FFT patterns, it is indicated that the G-phase maintains a cube-to-cube relationship with δ-ferrite. This is in agreement with previous reports on stainless steel welds and duplex stainless steels, attributed to the smaller mismatch in the lattice parameter with ferrite^6,8,10,46,47^.

After the R-HT, the coherent G-phase were still present in δ-ferrite for ER316L and ER347 welds (Figs 5c and 6c). Also, the size of the G-phase remained unaltered after the R-HT, similar to the previous report on duplex stainless steel^14^. In addition, a few occurrences of other intermetallic precipitate were observed for ER316L weld in R-HT condition (Fig. 5d), with FFT pattern matching with chi-phase (stoichiometrically Cr_8_Fe_18_Mo_5_). Similarly, formation of Mo-rich precipitates was reported in δ-ferrite in Mo-containing cast austenitic stainless steel during the R-HT^16^. In short, the δ-ferrite matrix after thermal aging consisted of phase decomposed δ-ferrite with G-phase while R-HT dissolved the phase decomposition.

### Electrochemical analysis of bulk specimens of welds

During the DL-EPR analysis, the Fe-rich α phase depleted with Cr will be attacked upon the reverse reactivation scan by the aggressive DL-EPR solution. Previously, Chandra *et al*.^6^ had performed DL-EPR analysis of the aged 316L weld in a concentrated solution (1 M H_2_SO_4_ + 0.1 M KSCN) and observed the reactivation peaks. However, we could not observe any reactivation peaks analysis performed in such solution for 316 L weld thermally aged at 400 °C. After several trials, we chose to use a more aggressive solution (2 M H_2_SO_4_ + 1 M KSCN) for ER316L weld. Meanwhile, Chandra’s solution (1 M H_2_SO_4_ + 0.1 M KSCN) was used for ER347 weld.

**Figure 7:**
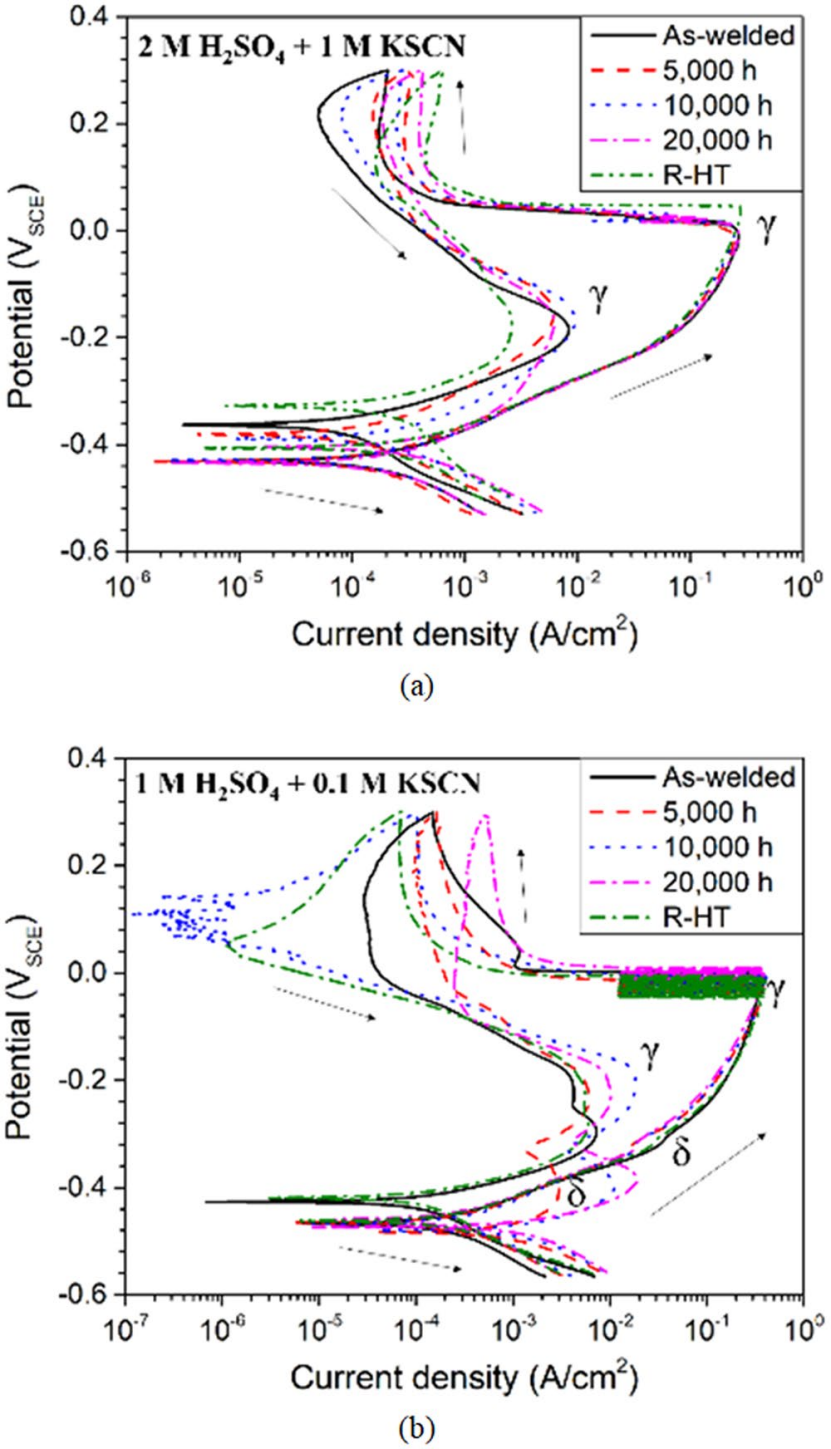
DL-EPR analysis of (**a**) ER316L (using 2 M H_2_SO_4_ + 1 M KSCN solution), and (**b**) ER347 (using 1 M H_2_SO_4_ + 0.1 M KSCN solution) welds in as-welded, aged and R-HT conditions.

The DL-EPR plots of ER316L and ER347 welds containing both austenite and δ-ferrite phases are shown in Fig. 7, and the measured current densities are summarized in Tables 3 and 4. Only a single activation peak (around 0 mV_SCE_) and reactivation peak was observed for ER316L weld in all conditions (Fig. 7a), which is similar to the electrochemical response of fully austenitic stainless steels. In addition, for ER347 weld, the activation peak corresponding to δ-ferrite was also not clearly observed during the forward scan, though a distinctive reactivation peak appeared during reverse scan (Fig. 7b). Generally, stainless steels containing both austenite and ferrite phases are known to exhibit two separate anodic dissolution peaks corresponding to each phase in the potentiodynamic polarization curves^41–43^. That is, the anodic peaks observed at lower potentials would be associated with the ferrite phase, while those at higher potentials with the austenite phase. Nevertheless, the absence of activation peaks of δ-ferrite for both welds can be attributed to the small amount of δ-ferrite phase (10 vol.%) in the welds, which will be verified later.

**Table 3:** Summary of the DL-EPR results of ER316L weld.

Condition	Current density of Peaks #1		
	i_a_ (mA/cm^2^)	i_r_ (mA/cm^2^)	i_r_/i_a_ × 100
As- welded	271.7	8.36	3.08
400 °C for 5,000 h	244.4	6.17	2.52
400 °C for 10,000 h	248.6	9.48	3.81
400 °C for 20,000 h	270.0	6.20	2.30
400 °C for 20,000 h + R-HT	283.6	2.61	0.92

**Table 4:** Summary of the DL-EPR results of ER347 weld.

Condition	Current density of Peaks #1			Current density of Peaks #2	
	i_a_ (mA/cm^2^)	i_r_ (mA/cm^2^)	i_r_/i_a_ × 100	i_a_ (mA/cm^2^)	i_r_ (mA/cm^2^)
As- welded	361.7	4.25	1.18	—	7.25
400 °C for 5,000 h	397.4	6.13	1.54	—	3.00
400 °C for 10,000 h	425.9	18.43	4.33	—	11.25
400 °C for 20,000 h	398.3	10.21	2.56	—	19.14
400 °C for 20,000 h + R-HT	416.4	5.56	1.34	—	5.98

For ER316L weld, no significant changes in both activation and reactivation peaks were observed after thermal aging as shown in Fig. 7a, though highly aggressive solution was used for the DL-EPR analysis. Meanwhile, for aged ER347 weld, the reactivation peak current density corresponding to the δ-ferrite and passive current density increased with aging time. This increase in reactivation peak current density can be associated with the Cr-depletion due to the spinodal decomposition in aged ER347 weld. Nevertheless, it must be noted that the observed reactivation peak current density values of δ-ferrite includes austenite electrochemical response to some extent. Therefore, the extent of microstructural changes in δ-ferrite cannot be estimated with the DL-EPR analysis on welds.

Meanwhile in previous DL-EPR studies of welds^6,7^, the electrochemical behavior after thermal aging were estimated based on the austenite peaks, which would be inaccurate to estimate the spinodal decomposition. Also in most cases^7^, no reactivation peaks corresponding to the spinodal decomposition in δ-ferrite appeared, indicating the overall electrochemical response was dominated by the austenite matrix. Therefore, the electrochemical analysis of δ-ferrite phase only was performed as an attempt to directly correlate with the microstructural changes in δ-ferrite.

### Selective etching of austenite matrix and specimen masking

**Figure 8:**
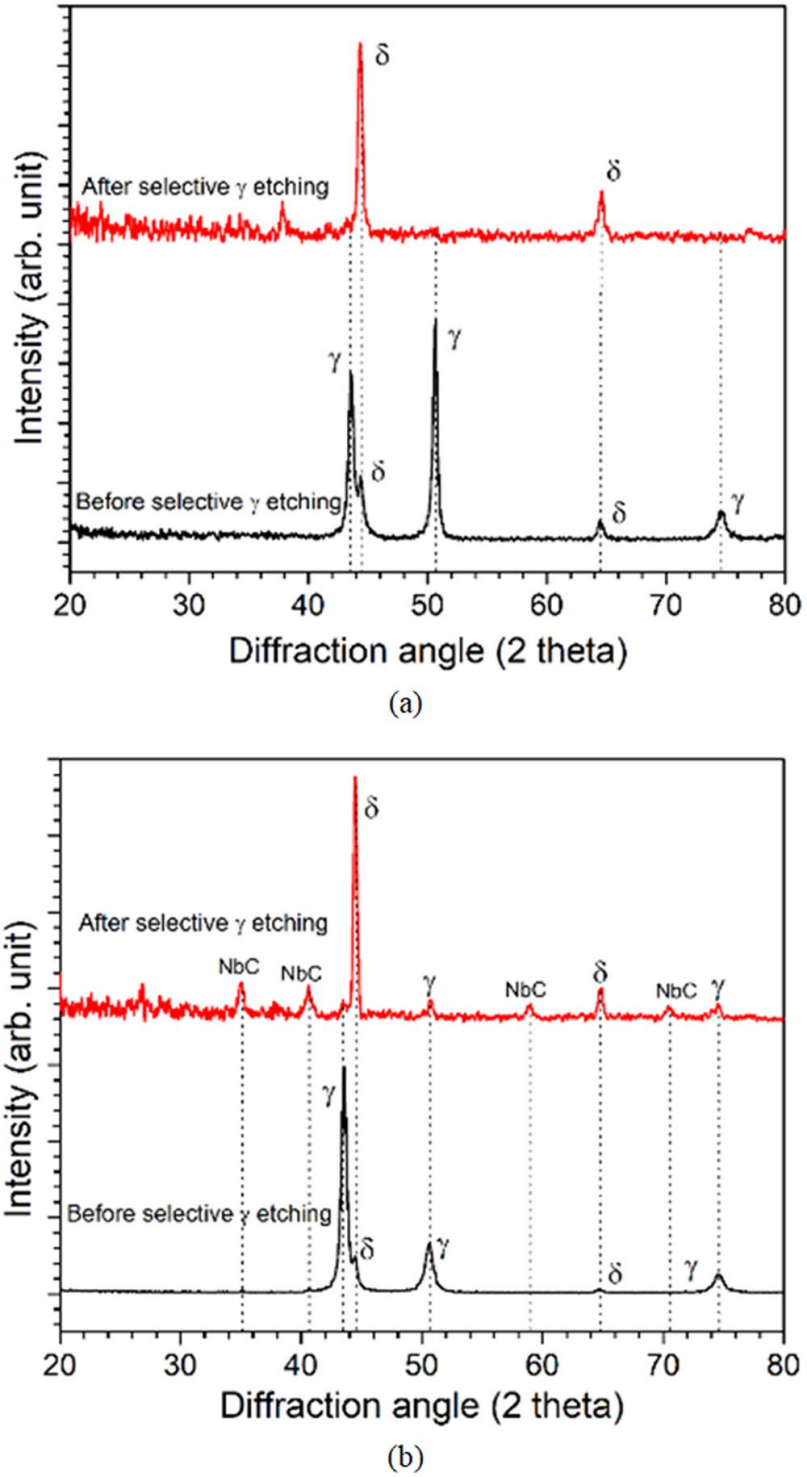
XRD results of (**a**) ER316L, and (**b**) ER347 as-welded specimen before and after selective austenite etching.

Örnek *et al*. demonstrated that the ferrite phase acts as anodic sites when galvanically coupled with austenite phase, indicating lower dissolution potential for ferrite phase^34^. We potentiostatically maintained at higher potentials where the austenite matrix dissolved out, while δ-ferrite remains unattacked. Figure 8 shows the XRD analysis results of ER316L and ER347 weld specimens before and after selective etching of austenite matrix. The strong austenite diffraction peaks observed in the as-welded specimens were effectively disappeared after selective etching of austenite matrix, leaving only δ-ferrite peaks. For ER347 weld, minor peaks of NbC also appeared along with some weak austenite peaks, which would have appeared from the bulk matrix beneath. It should be noted that the lack of NbC peaks in the as-welded ER347 weld does not suggest the absence of it, rather it is due to the very small volume fraction of NbC in the weld.

**Figure 9:**
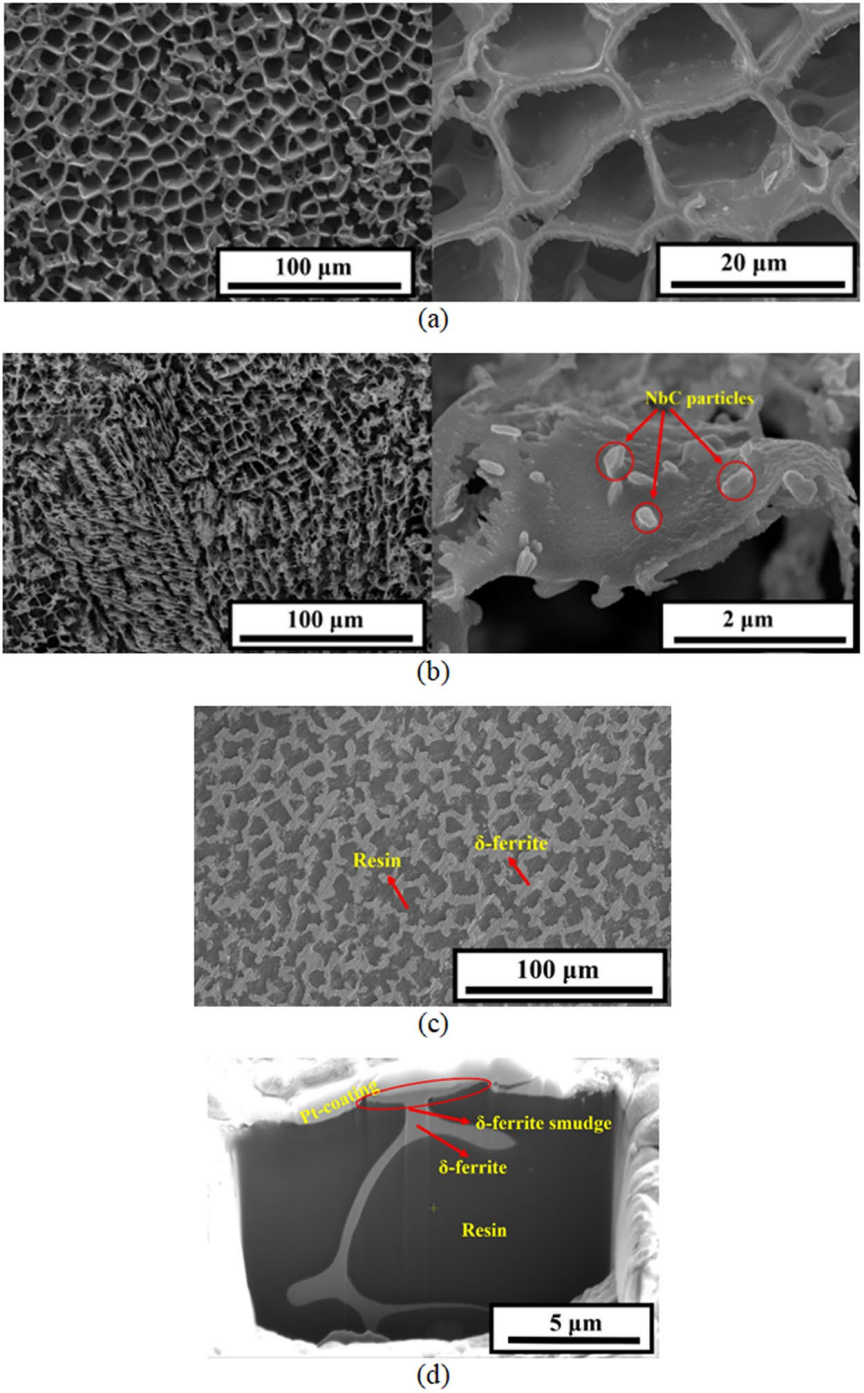
SEM images of (**a**) ER316L, (**b**) ER347 as-welded specimen after selective austenite etching, (**c**) surface, and (**d**) cross-sectional images of ER316L as-welded specimen after selective austenite etching followed by cold-setting epoxy resin filling and polishing.

SEM observation on the selectively etched specimens (Fig. 9) confirms the presence of predominant δ-ferrite microstructure with vacant cavities formed by the dissolution of austenite matrix. For ER316L weld (Fig. 9a), well developed honeycomb network of δ-ferrite was generally observed. In the case of ER347 weld (Fig. 9b), a combination of both vermicular and lacy morphologies of δ-ferrite were observed. At higher magnifications, small NbC particles were also found on the surface of δ-ferrite in ER347 weld, corresponding to the minor peaks in XRD results shown in Fig. 9b. It seems that the NbC particles at the δ-ferrite/austenite phase boundaries were not attacked during selective etching of austenite matrix. The austenite matrix was etched out to a depth of 40–45 μm for both the welds. After selective etching of austenite matrix, the vacant cavities were masked with cold setting epoxy resin, dried, ground and polished, to prepare the specimens for the electrochemical analysis of δ-ferrite phase, which is shown in Fig. 9c. Compared to Fig. 9a, the δ-ferrite appeared wider on the surface of the specimen. In fact, the δ-ferrite phase was found flattened, as shown in the cross-sectional SEM images (Fig. 9d), which may have been the results of inevitable damage during the specimen preparation like grinding and polishing. Nevertheless, as the flattening happened for all masked specimens and caused by local deformation, we may use the flattened specimens to evaluate and compare the electrochemical response of δ-ferrite due to thermal aging.

There were concerns of Cr-depleted regions in aged δ-ferrite phase being attacked during selective austenite etching. However, we did not observe any differences in δ-ferrite microstructure between as-welded and aged specimens after selective austenite etching, though not shown here. Since the δ-ferrite was ground after selective austenite etching, the passive film formed were removed from the surface, exposing the δ-ferrite microstructure for electrochemical analysis. Recently, we prepared nanopillars on the δ-ferrite after selective austenite etching for aged specimens^9^, and we found that microstructural evolution after thermal aging was maintained on the δ-ferrite structure as indicated by nanopillar compression test.

### Electrochemical analysis of δ-ferrite phase

**Figure 10:**
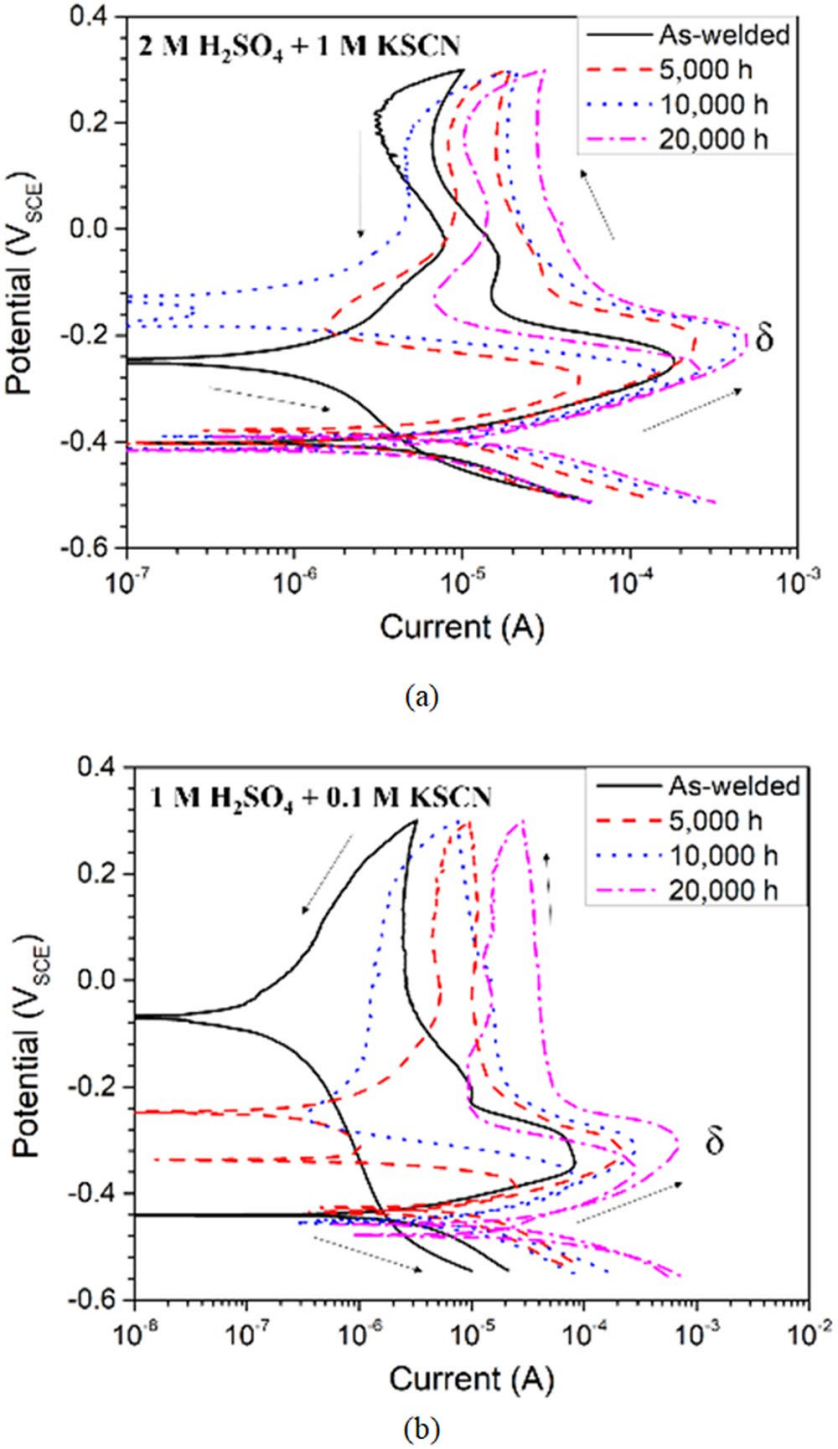
DL-EPR analysis of δ-ferrite from (**a**) ER316L (using 2 M H_2_SO_4_ + 1 M KSCN solution), and (**b**) ER347 (using 1 M H_2_SO_4_ + 0.1 M KSCN solution) welds in as-welded and aged conditions after selective austenite etching.

Figure 10 shows the DL-EPR plots of δ-ferrite phase from ER316L and ER347 welds measured for the masked specimens, and the measured current values are summarized in Tables 5 and 6. The anodic dissolution peak during forward activation scan occurred around −210 to −240 mV_SCE_ and −300 to −330 mV_SCE_ for ER316L and ER347 welds respectively. The potentials corresponding to the activation peaks for the masked specimens were much lower than the potentials for anodic dissolution peaks from the bulk weld material (around 0 mV_SCE_ as shown in Fig. 7). As explained earlier, the anodic dissolution peaks at lower potential in Fig. 10 is attributed to the electrochemical response from δ-ferrite phase, while the higher potential in Fig. 7 from predominant austenite matrix in bulk weld material. Therefore, it is confirmed that the absence of the activation peaks of δ-ferrite in the DL-EPR results of both welds (Fig. 7) was due to the small amount of δ-ferrite phase (10 vol.%) in the weld specimens.

**Table 5:** Summary of the DL-EPR results of δ-ferrite in ER316L weld.

Condition	Current		I_r_/I_a_ × 100		
	I_a_ (μA)	I_r_ (μA)		Average	Standard deviation
As- welded	114.3	—	—	—	—
	183.4	—	—		
	80.45	—	—		
400 °C for 5,000 h	398.9	59.26	14.86	16.65	2.55
	398.9	59.26	14.86		
	273.3	41.26	15.10		
	302.2	53.96	17.86		
	245.6	50.61	20.61		
400 °C for 10,000 h	430.5	147.7	34.31	29.29	2.82
	359.4	104	28.94		
	417.1	127.2	30.50		
	421.7	119	28.22		
	274	74.56	27.21		
	345.1	91.64	26.55		
400 °C for 20,000 h	503	260.4	51.77	45.27	7.56
	575.9	271.1	47.07		
	433.5	160.3	36.98		
400 °C for 20,000 h + R-HT	328.7	—	—	—	—
	407.9	—	—		
	408.4	—	—

**Table 6:** Summary of the DL-EPR results of δ-ferrite in ER347 weld.

Condition	Current		I_r_/I_a_ × 100		
	I_a_ (μA)	I_r_ (μA)		Average	Standard deviation
As- welded	82.41	—	—	—	—
	135.1	—	—		
	108.8	—	—		
400 °C for 5,000 h	214.2	25.52	11.91	7.46	3.51
	225.7	19.39	8.59		
	426.7	18.53	4.34		
	531.3	26.50	4.99		
400 °C for 10,000 h	189.4	59.42	31.37	29.27	1.91
	283.9	82.65	29.11		
	403.7	107.30	26.58		
	306.1	87.23	28.50		
	580	178.50	30.78		
400 °C for 20,000 h	682.3	284.10	41.64	39.81	2.28
	439.7	182.10	41.41		
	124.6	49.20	39.49		
	332.6	122.10	36.71		
400 °C for 20,000 h + R-HT	586.7	—	—	—	—
	176.0	—	—		
	113.6	—	—		

**Figure 11:**
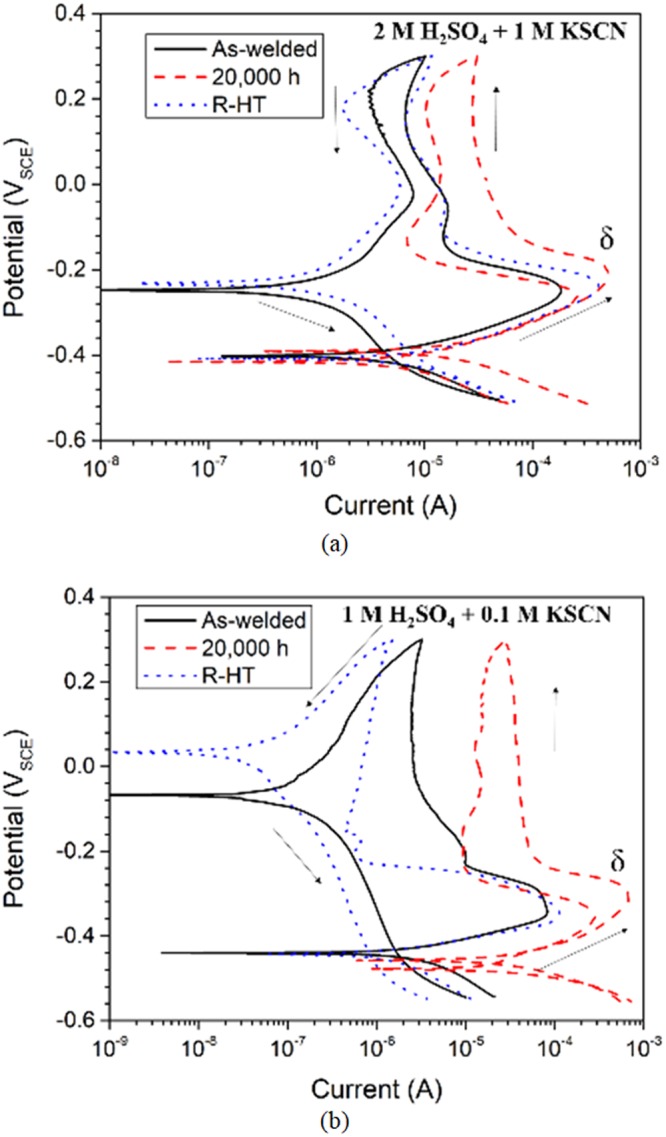
DL-EPR analysis of δ-ferrite from (**a**) ER316L (using 2 M H_2_SO_4_ + 1 M KSCN solution), and (**b**) ER347 (using 1 M H_2_SO_4_ + 0.1 M KSCN solution) welds in as-welded, aged at 400 °C for 20,000 h and R-HT conditions after selective austenite etching.

For the δ-ferrite phases in ER316L and ER347 welds, no reactivation peak was observed for the as-welded specimen (Fig. 10), indicating that uniformly distributed Cr provided superior corrosion resistance of the welds. However, reactivation peaks began to appear for the specimens thermally aged at 400 °C, and the corresponding current increased continuously with aging time, which can be associated with the formation of Cr-depleted regions in the aged specimens from spinodal decomposition, as shown in Figs 2 and 3. Also, DL-EPR analysis of δ-ferrite phase was conducted for R-HT specimens and the results are shown in Fig. 11. It is clear that reactivation peak was absent for the R-HT specimens of both ER316L and ER347 welds, and the overall shape of the DL-EPR curves was similar to that of the as-welded specimens. Therefore, the recovery of the spinodal decomposition by R-HT was confirmed in DL-EPR analysis. In other word, the reactivation peak current for the δ-ferrite in the aged welds can be only attributed to the formation of Cr-depleted α phase due to spinodal decomposition.

**Figure 12:**
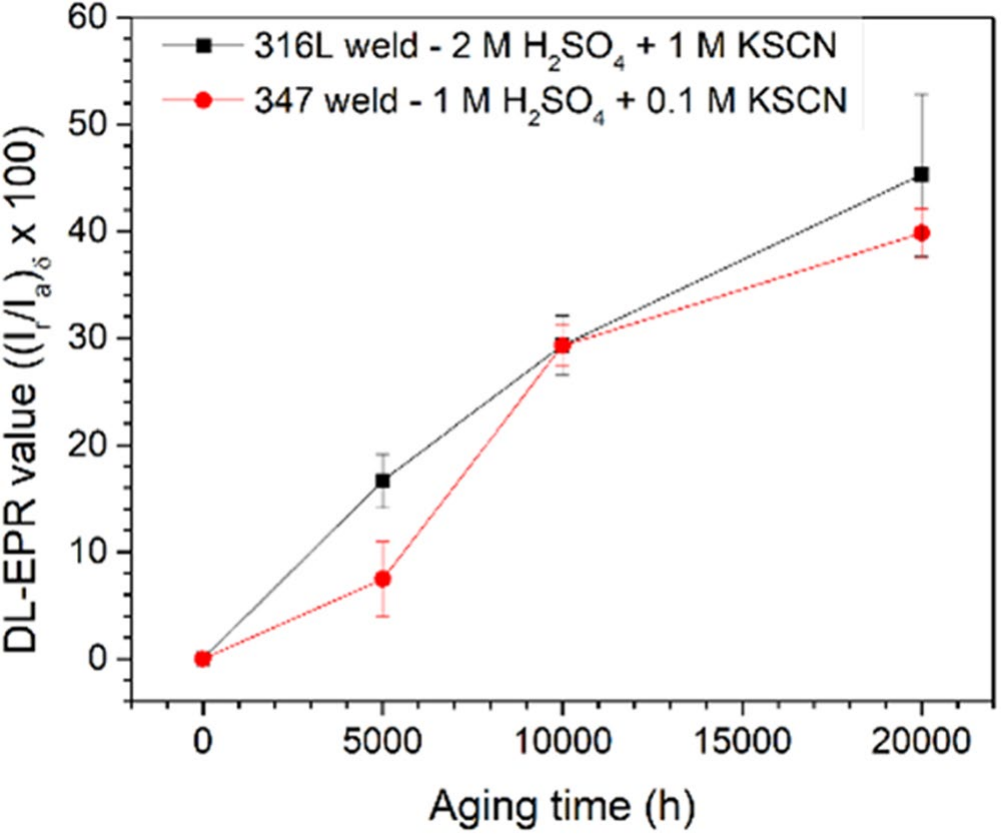
DL-EPR values of δ-ferrite from ER316L (using 2 M H_2_SO_4_ + 1 M KSCN solution), and ER347 (using 1 M H_2_SO_4_ + 0.1 M KSCN solution) welds in as-welded and aged conditions after selective austenite etching.

As mentioned previously, the surface area of δ-ferrite exposed in the DL-EPR analysis could not be accurately measured and thus the current density could not be calculated. Therefore, the degree of change in corrosion resistance of δ-ferrite due to thermal aging were estimated using the DL-EPR value of δ-ferrite ((I_r_/I_a_)_δ_ × 100), which was summarized in Tables 5 and 6 and plotted in Fig. 12 as a function of aging time at 400 °C. It should be noted that the electrochemical responses of ER316L and ER347 welds cannot be compared to each other as different solutions were used for each weld, as mentioned before. As shown in Fig. 12, the DL-EPR value of δ-ferrite steadily increases with aging time of up to 20,000 h for both welds indicating a continuous decrease in corrosion resistance of δ-ferrite. As the decrease in corrosion resistance is associated with local Cr-depletion with aging time, such observation would suggest that local Cr-depletion in α phase due to spinodal decomposition was yet to be saturated even after 20,000 h aging at 400 °C. However, the fluctuations in Cr content measured from STEM/EDS analysis are rather unchanged for the specimens aged at 400 °C for 5,000–20,000 h. The probable reason for this discrepancy would be the limitation of spectral resolution of STEM/EDS analysis, which failed to distinguish elemental variation in nano-scale.

It was reported that for the EPR analysis for sensitization studies of stainless steels, the electrochemical degree of sensitization (DOS) values increased when the minimum Cr-content present decreased to some critical value, typically around 10–13 wt.%^48,49^. The region with Cr-content below these critical values failed to form the protective passive film resulting in degradation of corrosion resistance. The DOS values increased significantly even for slighter reduction in Cr-content below this critical value. In addition, the DOS value was sensitive to the width of the Cr-depleted region. Similarly, the steady increase of DL-EPR values observed here can be attributed to the extensive depletion of Cr-content below a critical value necessary to form protective passive film and to the growth of Cr-depleted region. No self-healing phenomena is observed from the electrochemical analysis of δ-ferrite, which was previously observed in a few instances attributed to the replenishment of Cr into the Fe-rich α phase from the surroundings^6,24,25,33^. The steady increase in DL-EPR values of δ-ferrite indicate that the phase separation would not have yet reached final thermodynamic equilibrium. Though the kinetics of Cr-depletion will be sluggish at later stages, it would still result in significant changes to corrosion resistance of that region. In this regard, even negligible microstructural changes from spinodal decomposition can be effectively predicted using electrochemical analysis of δ-ferrite phase during the later stages of thermal aging.

On the other hand, the mechanical properties of the same materials used in this study showed elevated changes initially (5,000 h), while saturating at later stages (10,000 h). The extent of mechanical property degradation is almost concurrent with the microstructural changes from spinodal decomposition, in which both saturate at later stages. During later stages of phase separation, though negligible change is observed from microstructural perspective, it is still considerable for electrochemical behavior as the corrosion degradation intensifies below the critical Cr-content.

It can be observed that, the presence of intermetallic precipitates did not affect the corrosion resistance of welds. The Cr-content of G-phase precipitated in ferrite phase of aged stainless steels was around 10–20 at.% by APT analysis from other reported works^47,50–52^, lower than the average Cr-content in ferrite. Therefore, it would be unlikely for G-phase to deplete the Cr-content around and affecting the corrosion resistance of δ-ferrite. Meanwhile, the G-phase was reported to exhibit different dissolution potential in DL-EPR test^23^, and would not be attacked during δ-ferrite activation/reactivation reactions. Consequently, the changes in electrochemical behavior of δ-ferrite can be directly associated with the spinodal decomposition occurred during thermal aging.

As observed in Fig. 7, the electrochemical behavior of the bulk weld was dominated by predominant austenite matrix with minimal distinctive δ-ferrite response. Therefore, no proper correlations could be obtained between electrochemical behavior and microstructural segregation, though aggressive solution was used. Further, all the existing reports on welds^6–8,10,11^ studied the electrochemical behavior of the bulk weld dominated by austenite matrix, and would mean little correlation with the spinodal decomposition in δ-ferrite. On the contrary, the electrochemical analysis of δ-ferrite exclusively indicated increasing DL-EPR values with aging time, directly corresponding to the spinodal decomposition. Therefore, this method would more applicable to directly estimate the extent of spinodal decomposition in δ-ferrite phase than the usual bulk weld electrochemical analysis of thermally aged welds.

## Methods

### Weld materials and thermal aging

Blocks of welds were fabricated by building-up commercial grade welding wires of ER316L (Mo-containing low carbon grade) and ER347 (niobium stabilized grade) on 316L stainless steel plates by gas tungsten arc welding (GTAW) method. The compositions of weld wires were chosen in order to maintain δ-ferrite content of 10 vol.% in the finished welds. The chemical composition of as-welded materials was analyzed using inductively coupled plasma (ICP) method and the results are given in Table 7. Phase fraction of δ-ferrite content was determined by image analysis using ‘analySIS TS Material’ software from optical micrographs of the finished welds, and the results are also given in Table 7. Then the as-welded blocks were sectioned into small pieces and thermally aged at an accelerated aging condition of 400 °C in air environment for 5,000 h, 10,000 h and 20,000 h. A reversion heat treatment (R-HT) at 550 °C for 1 h was performed for both weld materials thermally aged at 400 °C for 20,000 h, in order to recover thermal aging embrittlement by dissolving the spinodal decomposition^14,16^.

**Table 7:** Chemical composition and ferrite content of welding blocks (in wt.%).

Weld	Fe	Cr	Ni	C	Si	Mn	Mo	Nb	Ferrite contents (vol.%)	
									Schaeffler diagram	Phase fraction
ER316L	Bal.	18.4	11.0	0.008	0.4	1.74	2.56	—	11	12.3 ± 1.3
ER347	Bal.	19.0	9.0	0.045	0.38	1.53	0.17	0.69	10	10.0 ± 0.4

### Microstructural analysis

For the microstructural analysis by transmission electron microscopy (TEM), thin foils were prepared from the weld blocks of the as-welded, aged, and R-HT conditions. The thin foils were mechanically ground to #1200 grit finish to thin down to 100 μm, followed by twin-jet electro-polishing in a solution of methanol and perchloric acid (9:1 ratio) at 20 V and –30 °C. Elemental mapping and line scan analysis was conducted using transmission electron microscopy equipped with energy dispersive spectrometer (STEM/EDS, Titan cubed G2 60–300) at 300 kV. High-resolution lattice images were taken using high-resolution transmission electron microscopy (HR-TEM, FEI Talos F200X) at 200 kV.

### Selective etching of the austenite matrix

To isolate δ-ferrite from the welds, selective etching of austenite matrix was used as follows. First, coupons with 3 mm in diameter and 2 mm in thickness were fabricated normal to TL (transverse-longitudinal) orientation from the weld material blocks in the as-welded, aged and R-HT conditions. The coupons were spot welded and cold mounted in such a way to expose the surface normal to the thickness direction, followed by grinding up to #4000 grit, and ultrasonic cleaning in ethanol. For selective austenite etching, a solution of 3.6 N H_2_SO_4_ + 0.1 N NH_4_SCN was used as described elsewhere^41,53^ with reference standard calomel electrode (SCE) and a Pt-wire as counter electrode. After several trials to find optimum condition to dissolve austenite phases, a potentiostatic condition of −120 mV_SCE_ was used for ER316L weld and −240 mV_SCE_ for ER347 weld, for duration of 1200 s. Gamry Reference 3000 potentiostat was used for all the electrochemical applications. The specimens after selective austenite dissolution was characterized using 2θ X-ray diffraction (XRD, Rigaku D/MAX-2500) and scanning electron microscopy (SEM, Hitachi model SU8230) at 10 kV.

### Electrochemical analysis

After the selective etching of austenite matrix, the etch pits were filled up with cold setting epoxy resin to prepare specimens for electrochemical analysis. The specimens were then dried, ground to #4,000 grit and ultrasonically cleaned in ethanol. The cross-sectional images after masking the specimen were taken using SEM equipped with focused ion beam (FIB-SEM, FEI model Helios G4) at 5 kV. DL-EPR analysis was conducted on the masked specimens revealing only δ-ferrite phase in the as-welded, aged, and R-HT conditions. Also, DL-EPR analysis of the bulk welds (before selective austenite etching) was conducted for comparison. A concentrated solution of 1 M H_2_SO_4_ + 0.1 M KSCN was used for ER347 weld, as previously reported^6,7^ to intensify the electrochemical response during reactivation scan. However for ER316L weld with better corrosion resistance, a more aggressive solution of 2 M H_2_SO_4_ + 1 M KSCN was used to observe the reactivation peaks during reactivation scan. A three-electrode system with the weld working electrode, a reference SCE and graphite counter electrode was used for the analysis. The specimens were initially maintained at a potentiostatic condition of –500 mV_SCE_ for 300 s to cathodically remove passive films on the surface, and then kept at free corrosion potential for 100 s. Then the potential was scanned from –100 mV_SCE_ below the free corrosion potential towards +300 mV_SCE_ at a scan rate of 1.66 mV/s, and then scanned in the reverse direction to the free corrosion potential. The DL-EPR analysis was repeated at least 3 times on different specimens to check the reproducibility. It should be noted that the current density in the DL-EPR analysis was not calculated since the surface area of the δ-ferrite phase in the specimen could not be accurately measured. In this case, the results of the DL-EPR analysis was quantified as the ratio of reactivation peak current (I_r_) to activation peak current (I_a_).

## Conclusions

1. Thermal aging caused elemental fluctuation by spinodal decomposition and G-phase precipitation in δ-ferrite. After R-HT, only the spinodal decomposition was removed.
2. After selective etching of the austenite phase, the DL-EPR analysis of the δ-ferrite phase showed steady increase in DL-EPR values with aging time up to 20,000 h, suggesting the continuous progress of Cr-depletion in α phase by spinodal decomposition.
3. Reactivation peaks were absent for the R-HT specimens of both ER316L and ER347 welds, indicating that intermetallic precipitates did not affect the corrosion resistance of welds. Therefore, the changes in electrochemical behavior of δ-ferrite can be directly associate with the spinodal decomposition.

## Data Availability

The datasets generated during and/or analyzed during the current study are available from the corresponding author on reasonable request.
